# Navigating the Challenges and Resilience in the Aftermath of the COVID-19 Pandemic in Adolescents with Chronic Diseases: A Scoping Review

**DOI:** 10.3390/children11091047

**Published:** 2024-08-27

**Authors:** Giovanna Cristina Machado-Kayzuka, Isabela Helena Seccarecio, Milena de Lucca, Rhyquelle Rhibna Neris, Ana Carolina Andrade Biaggi Leite, Willyane de Andrade Alvarenga, Paula Saud De Bortoli, Manoela Henriques Pinto, Lucila Castanheira Nascimento

**Affiliations:** 1 Ribeirão Preto College of Nursing, University of São Paulo, Ribeirão Preto 14040-902, Brazil; iseccarecioenf1@usp.br (I.H.S.); milenalucca@usp.br (M.d.L.); rhyquelle@usp.br (R.R.N.); paula.bortoli@usp.br (P.S.D.B.); lucila@eerp.usp.br (L.C.N.); 2 Departament of Health Sciences, Public University of Navarre (Upna), 31006 Pamplona, Spain; anacarolina.andrade@unavarra.es; 3 College of Nursing, Centro Universitário Santo Agostinho (UNIFSA), Teresina 64019-625, Brazil; willyalvarenga@unifsa.com.br; 4 Faculdade de Medicina de São José do Rio Preto, São José do Rio Preto 15090-000, Brazil; manoela.pinto@edu.famerp.br

**Keywords:** COVID-19, adolescent, chronic disease, family, physical distancing

## Abstract

Background/Objectives: The COVID-19 pandemic has profoundly affected the lives of adolescents worldwide, especially those living with chronic diseases. This study aims to explore the impact of the COVID-19 pandemic on the daily lives of adolescents with chronic diseases. Methods: This is a scoping review that follows the guidelines proposed by JBI. Eligibility criteria include articles focusing on adolescents aged 10 to 19 during the COVID-19 pandemic, regardless of chronic diseases. Searches were performed in PUBMED, LILACS, CINAHL, SCOPUS, grey literature, and manual searches in March 2024. Results: This review is composed of 35 articles. The analysis revealed two main categories: (1) Adolescents facing social isolation, school closure, and new family interactions, striving to reinvent themselves, and (2) Chasing the best decision: following up the chronic disease while fighting the COVID-19 pandemic. These categories encompass subcategories highlighting changes in social and family interactions and lifestyle habits. The findings suggest a multifaceted interaction of factors influencing adolescents’ well-being, including improved family bonding, heightened disease management, and increased stress and strains on resources. Conclusions: This review emphasizes the importance of long-term follow-up and social inclusion efforts for adolescents with chronic diseases and their families, addressing their unique needs during public health crises.

## 1. Introduction

The COVID-19 pandemic is emblematic of a modern public health crisis that has led to several changes in the world [1]. Actions to prevent the spread of the virus were adopted, and people’s daily lives have changed significantly [2]. Social distancing, school closures, the adoption of remote learning, and closures of outdoor recreational spaces and non-essential businesses were restrictions that inevitably affected the behavior, development, and well-being of children and their parents [3,4,5].

Although healthy children and adolescents are believed to be less susceptible to developing the severe form of the disease, those with chronic illnesses are characterized as a high-risk group for COVID-19 [6,7,8,9]. Chronic diseases (CD), defined as conditions that require continuous medical attention and have prolonged or indefinite durations, amplify the vulnerabilities of this population [10]. Adolescents with chronic illnesses are particularly vulnerable; the combination of their ongoing health needs and the restrictions imposed by the pandemic intensifies their dependence on caregivers, increasing the sense of uncertainty and the challenges of adapting to this new reality [11,12]. The caregiving burden borne by families of adolescents with chronic conditions poses multifaceted challenges, influencing familial dynamics and functioning [11,12,13].

Evidence shows that the pandemic has disproportionately affected the health of individuals under 18 and those with pre-existing health conditions, exacerbating existing disparities in healthcare access and outcomes, such as racial and economic ones [8]. Especially among adolescents, the period is marked by increased socialization and peer interaction, confronting a paradox of imposed isolation undermining developmental autonomy. This paradox is exacerbated for adolescents grappling with chronic diseases, mainly due to their dependency on parental support [14]. Because of treatment limitations and restricted follow-up procedures, these consequences can significantly affect the post-pandemic scenario. To restore healthcare continuity and enhance its effectiveness, it is crucial to pinpoint and comprehend the specific areas that require attention within this population [12,13,15]. While several studies have addressed the potential impacts of the COVID-19 pandemic on adolescents, to our knowledge, no review has been found in the literature that gathers evidence about the effects of COVID-19 on adolescents with chronic diseases. In this context, understanding the repercussions of the COVID-19 pandemic on the daily lives of adolescents with chronic diseases can fill the gap in scientific knowledge. It can also provide information for health professionals to promote evidence-based care that meets the specific needs of this population [15,16]. Consequently, this review aims to explore the impacts of the COVID-19 pandemic on the daily lives of adolescents with chronic diseases. 

## 2. Materials and Methods

This is a scoping review, an increasingly common evidence synthesis used to answer broad research questions by mapping evidence from multiple sources [17]. Moreover, scoping review results are essential for identifying knowledge gaps that future research should fill and where additional research efforts are needed, particularly in contexts with limited financial resources for research investment [18,19].

The steps proposed by the Joanna Briggs Institute (JBI) were used to guide the development of this review: (I) Development of the research question; (II) Definition of inclusion criteria; (III) Development of search strategies; (IV) Screening and selection of studies; (V) Data extraction; (VI) Analysis of evidence; (VII) Presentation of results [20]. The checklist PRISMA-ScR (Preferred Reporting Items for Systematic Reviews and Meta-Analyses Extension for Scoping Reviews) was used to guide this review report [19]. This review was not previously registered.

### 2.1. Search Methods and Selection Criteria

The PCC tool was used as a guide to formulate the research question [20]. The P is for population—adolescents with chronic diseases, C is for concept—impacts on daily life, and C is for context—COVID-19 pandemic. The developed research question is the following: What are the impacts of the COVID-19 pandemic on the daily lives of adolescents with chronic illnesses? The PCC structure search strategy comprised keywords, descriptors, and Boolean operators AND and OR. The searches were developed in the PubMed, CINAHL, LILACS, and SCOPUS databases. Each search strategy was adapted to the particularities of the databases, and an example is presented in Supplementary File S1.

Gray literature searches were conducted using Google Scholar. In addition, a manual search of the reference list of included studies was performed. The search was conducted in March 2024. Considering the authors’ language skills, the search was limited to studies published in Portuguese, Spanish, and English. We included primary or secondary studies conducted from March 2020 to January 2024, employing any methodological design or gray literature that addressed the routine of adolescents aged 10 to 19 years, as defined by the World Health Organization [10], living with chronic diseases [21], such as asthma, cystic fibrosis, and diabetes, during the COVID-19 pandemic. The focus was on physical health conditions. Studies published before March 2020 were excluded as we aimed to focus on the period after the World Health Organization declared COVID-19 a pandemic in March 2020. Studies were excluded if they investigated the viewpoints of families or healthcare professionals, included both children and adolescents without providing separate analyses for each group, utilized data from hospital records only, consisted of opinion articles or expert consensus studies, and conference abstracts or proceedings.

### 2.2. Data Extraction and Analysis

Studies identified in the databases were screened to exclude duplicates and then exported to Rayyan^®^ to verify titles and abstracts. This screening process was carried out independently by two reviewers. Any selection conflicts were resolved in a meeting between the reviewers. In the next phase, studies were read independently by the two reviewers in full. Selection conflicts were resolved in meetings to obtain the final sample. During this process, the inter-rater reliability obtained by Cohen’s Kappa was 0.87, indicating an almost perfect agreement between reviewers [22].

Two reviewers performed data extraction from the included studies. A form developed by the authors was created, and the information retrieved consisted of author, year, country, and objective; study design and data collection; population, sample, and chronic diseases; and primary outcomes. A third reviewer reviewed the extracted data [17]. 

For data analysis, the extracted data were coded according to the repercussions observed in the studies and then organized into categories and their respective subcategories [15]. Subsequently, a third reviewer thoroughly reviewed the data. Data were presented through tables, figures, and narrative descriptions.

## 3. Results

**Figure 1:**
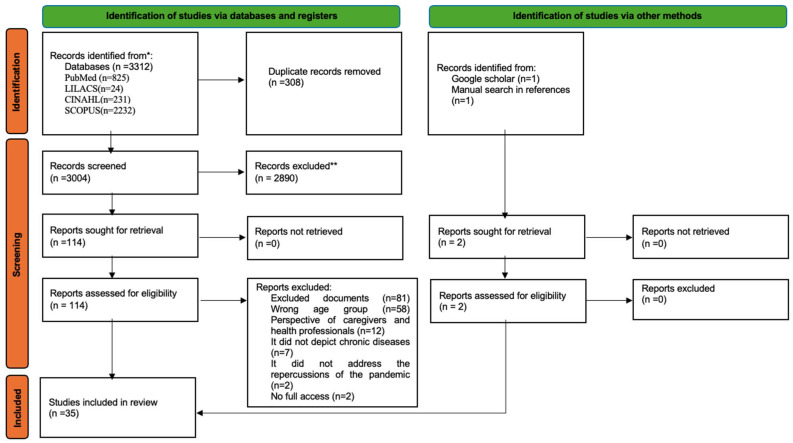
PRISMA flowchart of the screening process (Source: [23]). [color reproduction on the web]. * According to the search strategy; ** Records excluded during the title/abstract screening phase.

The PRISMA flow diagram (Figure 1) shows the studies’ selection process through the review [23]. From 3312 articles, 35 were selected for the final sample. 

### 3.1. Characteristics of the Included Studies

Table 1 provides a comprehensive overview of the critical characteristics of the articles analyzed in this study. The research was conducted across several regions worldwide, with a notable concentration in Europe (*n* = 13; 37.1%), followed by the Americas (*n* = 12; 34.2%) and the Middle East (*n* = 7; 20%). Additionally, a smaller number of studies were carried out in Asia (*n* = 1; 2.9%), Africa (*n* = 1; 2.9%), and Oceania (*n* = 1; 2.9%).

In general, the studies aimed to assess the adolescents’ psychosocial function and their quality of life (*n* = 29; 82.8%), the ongoing chronic disease follow-up in the COVID-19 pandemic (*n* = 15; 42.8%), and the adherence to COVID-19 preventive measures (*n* = 5; 14.3%). Six studies (17.1%) approached disparities in healthcare access and outcomes, although only one of them (2.8%) addressed the influences of racial issues on this aspect. Regarding study designs, 25 were quantitative (71.4%), 5 were qualitative (14.3%), and 5 used mixed methods (14.3%). The sample predominantly consisted of adolescents with type 1 or type 2 diabetes (*n* = 17; 48.6%), followed by those with intestinal inflammatory diseases (*n* = 5; 14.3%) and asthma (*n* = 5; 14.3%). Other diagnoses, such as cystic fibrosis, cancer, and heart/kidney diseases, were also represented in the sample. Eleven studies (31.4%) included parents’ perspectives or patients of different ages. Their results, however, allowed us to extract and analyze data related explicitly to adolescents independently. 

### 3.2. Categories and Subcategories

The main types of impacts were triggered mainly by the isolation from peers and families’ dynamics during the adjustment to the pandemic period, as represented in Table 2. The impacts described in the table were diverse and depended on various related aspects, which affected each family differently. Two categories and their respective subcategories summarize the findings: (1) Reinventing themselves when facing social isolation, school closure, and new family interactions and (2) Chasing for the best decision: following up the chronic disease while fighting the COVID-19 pandemic. 

#### 3.2.1. Category 1—Reinventing Themselves When Facing Social Isolation, School Closure, and New Family Interactions

This category comprises three subcategories: (a) Changes in social and family interactions, (b) Educational adaptations and the use of technology, and (c) Psychological and emotional changes.

(a) Changes in social and family interactions: The COVID-19 pandemic brought about significant changes in the family and social interactions of adolescents with chronic diseases [25,51]. With the need to stay home, these adolescents spent more time with their families. This increased presence of parents in the household had a dual impact [25,29,30,53]. Some studies reported that this increased time at home was associated with higher satisfaction among adolescents regarding their daily interactions with their families [47,53]. However, other research indicated that for some adolescents, the prolonged time spent at home was linked to higher levels of physical and psychological violence from their families [16,33,36]. These negative experiences were often underestimated and adversely affected the emotional well-being of those involved [16,33,36]. Furthermore, economic concerns further exacerbated the situation, as some parents left their in-person work due to fear of transmitting the virus to their children, resulting in worsened economic conditions and increased family tension [27,29,34]. The absence of parental presence at home was linked to worsened psychosocial well-being among adolescents, highlighting the complex dynamics that emerged during this period [39].

Isolation from society, including extended family and friends, contributed to increased psychological stress within the core family [39]. Adolescents faced the added challenge of stigma associated with their underlying conditions, such as cystic fibrosis, mainly due to the association between its symptoms and the symptoms of COVID-19 [44]. This led to increased stress for both parents and adolescents. In this context, social support from external networks was limited, and the disruption to social norms caused by the pandemic had a significant psychological and emotional impact on these adolescents [33,44].

(b) Educational adaptations and the use of technology: In addressing these challenges, remote learning emerged as an alternative to continuing education. Interestingly, adolescents adapted well to the transition to the online environment, benefiting their psychosocial functioning through social media connections [39,47,51,53]. However, concerns regarding the quality of education and safety in traditional settings persisted, contributing to resistance toward returning to in-person learning [26,33,34].

(c) Psychological and emotional changes: The psychological and emotional repercussions of social isolation were multifaceted. As it reduced their fear of being infected with COVID-19, contributing to making adolescents with chronic diseases less psychologically affected than their health peers [36,39,40,52,56], it also led to adverse effects such as increased sedentary behavior, anxiety, and stress [16,27,41,50,52,54]. The pandemic created uncertainty about the future, mainly due to the lack of information regarding its effects on chronic disease, jeopardizing financial and educational matters [33,38,47]. Mental health and overall quality of life of this population were also affected, with a noted gender difference in these experiences throughout the pandemic, with girls being more affected than boys [33,37,38,39,56].

#### 3.2.2. Category 2—Chasing for the Best Decision: Following Up on the Chronic Disease While Fighting the COVID-19 Pandemic

Three subcategories compose this category: (a) Influence of socioeconomic status on access to health services, (b) Changes in lifestyle habits and their consequences, and (c) New usual care routine related to chronic diseases.

(a) Influence of socioeconomic status on access to health services: The COVID-19 pandemic profoundly impacted healthcare services available to adolescents, particularly those from diverse socioeconomic backgrounds [24]. Adolescents relying solely on public healthcare services faced exacerbated challenges in managing chronic diseases due to the reallocation of resources to attend to emergent populations during COVID-19 [24,34]. Access to vital healthcare resources, including continuous monitoring devices for diabetes, became limited, resulting in worsened disease control and symptoms [29,45]. Conversely, adolescents with private medical–hospital insurance experienced improved disease management through telemedicine and maintained access to control devices, leading to better outcomes [24,28,29,32,41,49].

(b) New care routine related to chronic diseases: The pandemic’s disruptive effects extended beyond healthcare services and entered adolescents’ daily lives. Socioeconomic vulnerabilities, such as housing insecurity and income instability, impeded their capacity to manage chronic diseases effectively [28,29,43,49]. Fears of contracting COVID-19 deterred adolescents and their families from seeking in-person medical follow-up, leading to decreased healthcare continuity [26,51]. Misinformation about COVID-19 and its interaction with specific medications for the usual treatment of the chronic disease led some adolescents to discontinue their treatments [26,27].

Adolescents who had parental supervision at home supporting the disease management adhered more readily to treatment due to the maintenance or increase in monitoring of signs and symptoms [24,25,29,30,43,46,48,53,54]. Adolescents’ belief that chronic diseases could be a risk factor for a worse COVID-19 prognosis also contributed to better disease control [30]. On the other hand, routine for chronic disease control, such as monitoring and testing frequency, worsened when adolescents self-managed their care without parental supervision [28,32,37,45].

(c) Changes in lifestyle habits and their consequences: The adoption of self-management practices emerged as a potential solution to control chronic diseases, improving adherence to treatment and overall health [42,52,54,55]. Maintaining physical activity levels, practicing healthy nutrition, including participating in home-cooked meals, and adapting to new routines were crucial in promoting well-being [15,30,39,40,45,46,53,54]. However, prolonged isolation had adverse effects on lifestyle habits, leading to changes in sleep patterns, increased sedentary behavior, changes in eating patterns, and even the emergence of eating disorders [15,27,28,32,35,38,39,52,54]. There was a notable reduction in physical activity among adolescents with chronic diseases, who demonstrated poorer physical health compared to healthy peers [14]. Adolescents adapted to some COVID-19 prevention measures, such as frequent handwashing [27,34,44,51,52], while encountering difficulties with others, like using hand sanitizers and masks, particularly those with chronic skin conditions [26,34].

**Figure 2:**
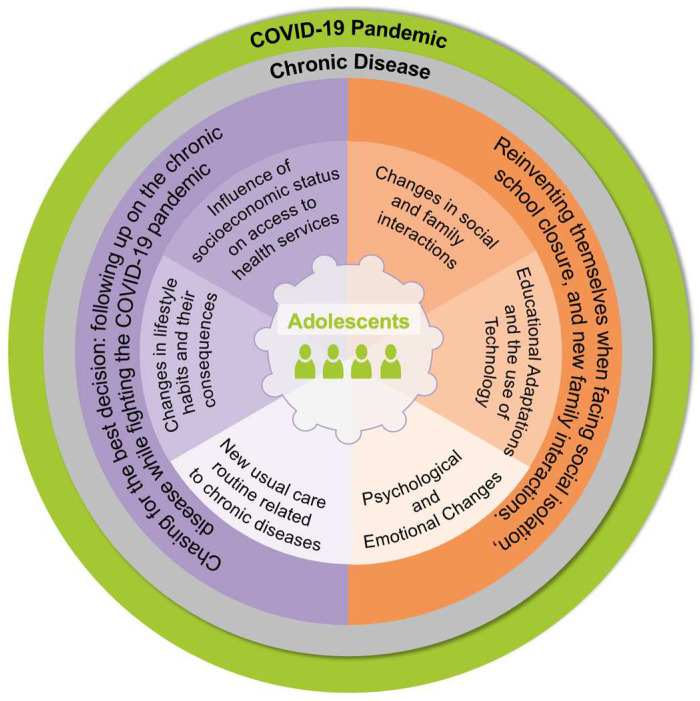
Representation of the impact of the pandemic on the daily life of adolescents with chronic illnesses (Source: Authors) [color reproduction on the web].

The impacts of the COVID-19 pandemic on the daily lives of adolescents with chronic diseases are represented in Figure 2.

## 4. Discussion

This review explored the impacts of the COVID-19 pandemic on the daily lives of adolescents with chronic diseases. Constrained in their homes due to social isolation, adolescents transformed their living spaces into new worlds. Within these households, fundamental needs such as social interaction, mental and physical well-being, and financial stability became closely intertwined with family needs. In this context, families expanded their protective role against the pandemic’s dangers. Adolescents and families had the opportunity to collaborate toward common goals during these challenging times, finding new solutions and making adjustments that acted as windows to navigate this new reality. However, if there was an imbalance between the needs of the family and the adolescent, the stability of this new world could be compromised, leading to potential setbacks.

The studies included were developed all over the world, illustrating the global impact of the pandemic. However, there was relatively limited evidence from Asia, Africa, and Oceania, which may be attributed to lower-income countries’ restrictions on research development, mainly COVID-19 and its impact on people with chronic diseases [57,58,59].

The results of this review show a strengthening of family bonds due to social isolation. Through greater parental vigilance, adolescents adopted healthy lifestyle habits, such as cooking and starting physical exercises at home, which benefited chronic disease control. However, the separation from their peers and the family tensions resulting from this increased coexistence aroused feelings of loneliness and insecurities linked to the vulnerabilities of their chronic disease. Similarly, scientific evidence in different contexts suggests that increased coexistence between the adolescent and the family established a new family dynamic that supported relationships and alliances between family members and presented a willingness for more effective communication [43].

The fear of coronavirus infection among this population was also shown in our results as impacting family dynamics and social relationships. In this context, family isolation acts as a protection mechanism. The consistent presence of caregivers at home during the pandemic has also proven to facilitate the control of chronic disease symptoms, mainly due to the positive change in adolescents’ habits. Despite being evident during the pandemic, social isolation is already a frequent topic in studies with families of people diagnosed with chronic diseases, as caring for their loved ones becomes a priority of daily living [60,61]. In family functioning, activities and routines such as eating, sleeping, and working are present in the lives of these families [42]. The diagnosis of chronic disease in the family and the new care routine significantly influence their daily activities [60,61].

The literature indicates that the unexpected disruption of the family routine caused by the pandemic can lead to increased stress for caregivers, mainly due to the interruption of work and the added responsibilities of other family members [57,62]. Our results indicated that concerns regarding chronic diseases and the fear of contamination by the coronavirus were triggers to isolation, becoming central daily concerns. The family increased control over the adolescents and their routines as a protection mechanism. Although it benefits health, excessive control contributes to increased interfamily stress. The family financial burden was another critical factor for the stress intensification in family relationships found in our study. These results may be associated with the observed global trend of a significant increase in unemployment rates, mainly due to the closure of companies during the most critical phase of COVID-19 transmission [63,64]. Furthermore, the loss of support from the extended family in care due to isolation and the performance of new roles in the family routine, including educational support, also makes sense for the increased negative feelings [65,66].

As found in our review, in addition to losing their jobs, many guardians chose to move away from the in-person work environment to avoid bringing the disease to their children. Other studies developed with the adult population showed a higher incidence of mental health-related problems throughout the pandemic period [67], significantly among patients whose economic conditions deteriorated. The loss of their caregivers’ jobs was the main factor, and those who faced hunger experienced higher levels of sadness or hopelessness [67]. This fact was also observed in a German study [68], in which the impact of the failing socioeconomic status affected their access to quality health services [64,68]. Thus, the need to choose between leaving their jobs to protect and care for the patient and the financial needs of families represents a continuous and essential decision-making process faced by families [61,62].

These changes and decisions were also found in our results, consolidating the scientific evidence that legitimizes the significant impact of the pandemic on increasing fear and distress among adolescents. While existing literature indicates that parents of adolescents in a typical chronic disease diagnosis process face increased psychological distress [69], our results showed that in the population included, some families may have found it easier to manage mental health during the COVID-19 pandemic compared to individuals without chronic diseases. The higher resilience of those families may justify this fact [61,70], developed by the adjustments in the family routine and family sacrifices, such as changes in employment status and the need for frequent medical follow-up, which families have known since the disease’s diagnosis [60,62].

Other differences were observed between healthy and diagnosed adolescents. The way adolescents with chronic diseases coped with the new routine, for example, was sometimes harmful. Two distinct contexts were experienced: the ability to overcome challenges through adaptations or the difficulty in overcoming the changes imposed by the new routine. Successful adaptations were represented by the excellent use of technologies in online teaching and health care modalities. Its use, however, also depended on the resources available to each family nucleus. Such resources were understood as material resources and systems of communication, organization, and family beliefs. These factors were also crucial in other contexts, including family systems, directly influencing their ability to deal with new challenges and barriers [61,69].

The difficulties in adapting were primarily related to stress, which made it difficult to reestablish the equilibrium of the family system and identify their feelings and experiences. Some of the studies in this review pointed to a distance between the teenager and the family resulting from this difficulty. The lack of supervision regarding the care and lifestyle of adolescents was one of the main consequences of these conflicts. The increase in interfamily violence reported in our results also caused harm to the well-being and health of this population. These barriers may be justified by the changes brought about by the COVID-19 period, which reinforced an already deficient pattern of interactions, making it increasingly difficult for adolescents to feel understood within their needs [69,71]. Such negative aspects are also closely related to social isolation, influencing and being influenced by the economic, social, and psychological consequences discussed previously [64,65]. This dynamic emphasizes the relationship between the adolescent and the family involved in the disease process, affecting the family’s functioning and ability to deal with challenges [72].

Thus, through the synthesis of the studies found, it was evident that the presence of the family was perceived as a critical element for the pandemic impact on the daily lives of adolescents with chronic diseases. Parental surveillance was a protective factor for adopting healthier lifestyle habits and, consequently, adopting adaptations that allowed for maintaining and improving health conditions.

We could also identify both the strengths and limitations of our study. Notably, our investigation is a unique contribution to the literature, being up-to-date and offering a multi-faceted view of the various impacts of the pandemic on different aspects of the daily lives of adolescents with chronic illnesses, with no restrictions placed on their specific diagnosis. However, it is essential to acknowledge several limitations. Firstly, our focus was not on understanding mental health aspects, which is why related terms were not included in our search strategy. We recognize that this may have limited the comprehensiveness of our search. Additionally, the search was restricted to studies published in Spanish, English, and Portuguese, potentially excluding relevant research published in other languages. Nevertheless, this review was able to capture studies conducted in different countries. Although the original studies did not delve into the cultural and health contexts of the respective countries where they were conducted, it is important to highlight that the impact of the COVID-19 pandemic on the daily lives of adolescents with chronic diseases was influenced by the varying healthcare systems. Another notable limitation is that the majority of studies included in our review focused on adolescents with diabetes mellitus, with 17 out of 35 studies examining this particular condition. This highlights a significant gap in the research regarding other chronic diseases, such as cystic fibrosis, cancer, and heart/kidney diseases, which also affect adolescents and were impacted by the COVID-19 pandemic but have been less extensively studied.

### 4.1. Practical Implications

This study outlines challenges adolescents with chronic illnesses faced during the pandemic, including routine changes, healthcare access, and emotional well-being. Given the significant impact on psychological and emotional health identified in our findings, it is essential to prioritize the expansion of mental health services in response to future health crises. Enhancing access to psychological care can play a crucial role in alleviating the emotional strain experienced by these adolescents. These findings can guide professionals in supporting this population by identifying key areas of support and strategically allocating resources across diverse socioeconomic contexts.

### 4.2. Implications for Future Research

Future research should specifically focus on the dimensions of “psychosocial and emotional aspects” to gain a more nuanced understanding of these critical factors. By incorporating these elements into research strategies, studies can provide deeper insights into the mental health challenges faced by adolescents with chronic conditions during the COVID-19 pandemic. This targeted approach will enhance our understanding of the full impact of the pandemic on this vulnerable population and inform more effective interventions and support mechanisms. Additionally, future studies should also aim to explore how disparities in access and outcomes of healthcare services that already existed between people of color and white individuals were exacerbated by the pandemic. Addressing this gap could provide a more comprehensive understanding of the factors influencing health inequalities and guide strategies to mitigate such disparities in future public health crises.

## 5. Conclusions

The study explored how the COVID-19 pandemic affected adolescents with chronic diseases, highlighting its global implications. Due to social isolation, family bonds were strengthened, leading to healthier habits and better disease control through parental vigilance. However, separation from peers caused loneliness and insecurity, exacerbating their vulnerabilities. Increased family cohesion improved communication but also heightened concerns about coronavirus infection. Being isolated was a protective mechanism, but it also contributed to increased interfamily stress and financial burdens. Adolescents faced more significant stress due to disrupted routines and economic strain, which impacted family dynamics. Despite these challenges, the ability to adapt to changes and prioritize mental health contributed to their resilience. Successful adaptation depended on available resources and family beliefs. Overall, the study emphasizes the crucial role of family support in mitigating the pandemic’s impact on adolescents with chronic diseases, highlighting the importance of parental supervision in fostering healthier lifestyles and coping mechanisms. Moving forward, addressing the unique needs of adolescents with chronic illnesses during crises is crucial to guiding practice and conversations with this population.

## Figures and Tables

**Table 1:** Characterization table of included studies. * Studies in which participants’ ages are marked with an “*” had only the results pertaining to the inclusion criteria (adolescents aged 10–19 years) analyzed.

Reference, Country, Objective	Study Design, Population, Sample, and Chronic Disease	Main Results
Abdulhussein et al., 2021 [24] United States of America To evaluate the effect of lockdown on glycemic metrics in youth with type 1 diabetes (T1D) using continuous glucose monitoring in northern California.	Retrospective cohort study. Measurement of blood glucose levels through continuous monitoring and the association between demographic variables (gender, age, and having or not having medical insurance) and changes in glycemic values, pre- and post-lockdown. Study population: 43 children aged 3 to 12 years old and 28 adolescents aged 13 to 18 years old diagnosed with T1D.	Participants with access to private health insurance reduced their average glucose levels between the pre- and post-lockdown periods. Those with public health insurance showed a decrease in the percentage of time at the target glycemic value and an increase in the time above the target. Between groups, children and adolescents showed reduced blood glucose variability compared to young adults (between 18 and 21 years old). No association was found between gender and blood glucose.
Çelik et al., 2022 [25] Turkey To investigate the psychosocial impact of the pandemic on pediatric patients with congenital adrenal hyperplasia and their families and whether congenital adrenal hyperplasia imposes an additional burden compared to other endocrine disorders.	Longitudinal study with a mixed approach. Daily routine scores, country, and children’s level of information about COVID-19 were calculated to assess the severity of changes in daily routines. Factors that interfere with medication adherence were also questioned. Depression and anxiety were assessed using scales. Study population: 79 children aged 6 to 18 years old * diagnosed with congenital adrenal hyperplasia or congenital hypothyroidism.	Increased levels of anxiety among parents and children, especially among adolescents over 12 years old diagnosed with congenital adrenal hyperplasia. The two diseases had similar scores related to daily routines and information about COVID-19. The main changes in the daily routine were time spent on school activities for the adrenal hypoplasia group and relationships with siblings in the hypothyroidism group. Regarding the level of information about COVID-19, the “risk of contracting the disease or having a worse diagnosis as a result of the chronic disease” was significant for children over 12 years old with adrenal hyperplasia; for patients with hypothyroidism, “prevention of Sars-Cov-2 infection” was the most significant item. For adherence to drug treatment, the presence of parents at home was the factor that most contributed to the success of treatment; changes in the pattern and duration of sleep impaired adherence to treatment.
Beytout et al., 2021 [26] France To investigate the impact of the COVID-19 pandemic on children with psoriasis in France and the impact of psoriasis on the ability of these patients to adhere to preventive social and health measures.	An exploratory survey study with a quantitative approach. A questionnaire was used to assess the impact of the pandemic and lockdown on children. The study evaluated the impact of psoriasis on children’s adaptation to COVID-19 preventive measures and return to school. Study population: 92 children and adolescents aged 7 to 15 years old diagnosed with psoriasis.	Ten patients abandoned the psoriasis treatment due to non-renewal of a medical prescription, the low impact of psoriasis on the adolescent’s life, their own decision supported by their parents, and fear of developing severe forms of COVID-19. Thirty-eight patients maintained medical appointments during the pandemic (twenty-seven via teleconsultation). The reasons for consultation were related to the worsening of psoriasis and a previously scheduled consultation. Twenty-one patients reported difficulty adhering to COVID-19 prevention measures due to psoriasis, mostly related to the application of 70% alcohol to the skin and the use of masks. Twenty-four adolescents reported having no plans to return to school due to the school still being closed, parents’ desire to keep their children at home, ongoing treatment for psoriasis, and worsening of the underlying disease and its association with other chronic diseases, such as asthma.
Cekic et al., 2021 [27] Turkey To assess the attitudes of adolescents with asthma toward the COVID-19 pandemic and determine the effects of the pandemic on their quality of life.	Quantitative study, using a questionnaire to assess compliance with protective measures against COVID-19. Measurement of concern about COVID-19, applying a visual scale with numbers between 0 and 10 to assess the level of concern. Assessment of quality of life through a scale. Study population: 25 adolescents aged 12 to 18 years old diagnosed with asthma and 98 healthy peers.	Twenty-two adolescents reported having given up on medical care, even with health complaints, mainly due to the belief that COVID-19 could be more easily transmitted to them. Regarding quality of life, participants with asthma had lower scores related to dissatisfaction with their health status, economic situation, and domestic conditions; however, they presented higher satisfaction scores with the performance of activities of daily living compared to the healthy group. Regarding protective measures against COVID-19, the group with asthma and the healthy group had similar rates. Regarding activities of daily living, patients with asthma showed higher rates of changes in their economic situation and increased use of vitamins and medications to prevent COVID-19. They also showed changes in sleep patterns and appetite, although to a lesser extent than in the healthy group; changes in sleep patterns were associated with fear of COVID-19 infection, and changes in appetite were linked to treatment abandonment. Asthma patients also reported having less contact with positive cases of COVID-19 and lower sadness scores for not leaving home than the healthy group. Girls had higher scores for fear of infection, sadness due to social isolation measures, and greater frequency of hand washing.
Cheng et al., 2021 [28] Malaysia To examine the impact of lockdown on glycemic control and lifestyle changes in children and adolescents with type 1 (T1D) and type 2 (T2D) diabetes mellitus under the age of 18 years.	Cross-sectional study with a mixed approach. Qualitative interviews were carried out on diabetes control and lifestyle changes before and during lockdown. Questionnaires were used to assess physical activities. Study population: 123 participants, 93 diagnosed with T1D, aged 7 to 15 years old *; 30 diagnosed with T2D, aged 10 to 18 years old.	Male adolescents over 13 had worse glycemic control, related to greater independence from adults and higher closeness to their peers. No significant changes in glycemic control were observed for patients with T1D. When compared, patients with T1D showed an increase in weight and BMI, and patients with T2D had a reduced BMI. There was a reduction in meal frequency in both groups. Those with low physical activity levels during the pandemic experienced a worsening pattern after the lockdown. Physical activity time decreased from 226 min to 213 min per week in patients with T1D, and physical activity time decreased from 229 min to 187 min per week in patients with T2D. There was an increase in screen time, between 2.75 and 5 times more than previously. An increase in sleep time was also observed. Most patients with T1D underwent medical follow-up through online consultations, but access was limited due to a lack of human resources, a lack of a dedicated app for consultations, and limited access to technology for some families.
Choudhary et al., 2022 [29] United States of America To examine the impact of the pandemic on hospitalization, clinic visits, glycemic control, and frequency of depression in children and adolescents with type 1 diabetes (T1D).	Retrospective study with a quantitative approach. Pre- and post-COVID-19 groups were evaluated. A questionnaire was applied to assess depression. Blood was collected to evaluate glycated hemoglobin and blood glucose values. Sociodemographic data were also collected. Study population: adolescents aged 10 to 18 years old diagnosed with T1D.	An increased incidence of diabetic ketoacidosis was observed in patients with T1D, along with an increase in glycated hemoglobin values, especially among patients without medical insurance and patients of Black race. There was difficulty in obtaining insulin among the poorest population. Continuous glucose monitors and insulin pumps have been associated with stable or improved glycemic control; however, this feature has been underutilized due to high costs. Telemedicine-assisted clinical visits and improved glucose control, mainly when using continuous glucose monitoring; however, there was low population adherence to this resource. Hospitalization frequencies remained unchanged during the pandemic despite decreased office visits among patients with insurance. Greater parental supervision made it possible to improve diabetes control.
Cusinato et al., 2021 [30] Italy To evaluate the impact of lockdown during the COVID-19 pandemic on glycemic control and psychological well-being in young people with type 1 diabetes (T1D).	Cohort study with a quantitative approach. Verification of blood glucose values assessed before and during the pandemic. A scale was used to assess the presence of anxiety and depression, exploring thoughts, feelings, and behaviors. Study population: 117 adolescents and young adults aged 12 to 20 years old *, diagnosed with T1D.	Patients increased the frequency of blood glucose measurements during the lockdown, leading to a reduction in episodes of hypoglycemia and hyperglycemia. Patients who presented anxiety and depression had less control of the disease. Adolescents with T1D improved their glycemic control by around 10%. As the pandemic continued, there was an increase in the presence of depression and anxiety and a consequent worsening of adolescents’ adherence to blood glucose control measures. Factors associated with the improvement in glycemic control included the inclusion of home-cooked meals, greater self-care, increased parental control, awareness that diabetes could worsen COVID-19, and a low prevalence of psychological disorders.
Dorfman et al., 2021 [31] Israel To assess the impact of COVID-19 on healthcare delivery, fear of infection, continuity of medical treatment, and compliance with preventive instructions in children and adolescents with inflammatory bowel disease.	Cross-sectional study with a mixed approach. A questionnaire was used to assess behavior and adherence to treatment and the impact of COVID-19. Study population: 244 children and adolescents between zero and 18 years old * with pediatric inflammatory bowel disease, such as Crohn’s disease and ulcerative colitis.	Forty-three participants perceived a worsening in the provision of health services. One hundred eighty-one reported no difference in the availability of medical treatment during the pandemic. One hundred sixteen patients reported not feeling comfortable going to the health service. One hundred seventy-eight patients said they were afraid to go to the emergency room in case of an exacerbation of the disease. Only seven patients discontinued drug treatment. One hundred ninety-eight reported fear of developing severe forms of COVID-19 due to their illness and treatment. Two hundred twenty-eight reported strictly following protective measures against COVID-19. Ninety-one reported not attending school as a form of prevention. Around 7% extended the protective measures to their families, including siblings prohibited from attending face-to-face classes, parental dismissal from work, and family social distancing. Most patients reported not receiving enough information about the effect of COVID-19 on their illness. Adolescents reported not strictly following protective measures against COVID-19.
Elhenawy; Eltonbaryl, 2021 [32] Egypt To evaluate the impact of the pandemic and lockdown on glycemic control among Egyptian children and adolescents with type 1 diabetes mellitus (T1D).	Cross-sectional study evaluating eating habits, diabetes care, and lifestyle habits before and after lockdown; treatment regimen, including the frequency of hypo- and hyperglycemia before and after the pandemic; results of high-performance liquid chromatography (HPLC) examination. Concerns related to COVID-19 were measured using a scale. Study population: 115 children and adolescents under 18 years old * diagnosed with T1D.	The results showed worsening eating habits and diet control. Overall, diabetes control was worse during lockdown in almost 59% of patients. Only 40% measured their blood glucose at least five times a day. Most patients reported more frequent episodes of altered blood glucose levels (hyperglycemia or hypoglycemia). There was an increase in insulin dose in 61.7% of patients. Glycated hemoglobin worsened in two-thirds of the population, mainly in adolescents. As for physical exercise, 18% of patients did some sport, and only 5% maintained physical activities at home during the pandemic. Most patients maintained medical follow-up through telemedicine, although 3% discontinued it due to access difficulties. Concerns related to the pandemic included fear of infection, fear of T1D exacerbation requiring hospitalization, and fear of cutting medical supplies.
Fedele et al., 2022 [33] Canada To assess health-related quality of life (HRQOL) in children affected by inflammatory bowel disease (IBD) during the first wave of the COVID-19 pandemic and after twelve months and determine which factors influence HRQoL in patients with IBD.	Prospective observational study with a quantitative approach. Application of instruments to evaluate the monitoring of the disease, anxiety, and quality of life. Participants were also asked whether they or a family member had contracted COVID-19 at any point. Study population: 118 patients aged 10 to 18 years old diagnosed with inflammatory bowel disease (Crohn’s disease or ulcerative colitis).	Body image and systemic symptoms presented the lowest quality of life scores, mainly among girls. IBD treatment and symptoms recorded the highest scores. After 12 months, a significant decrease in the median HRQoL was observed, particularly in IBD symptoms, emotional domain, body image, and treatment. Only 4 of the 118 adolescents had anxiety scores above average, with no changes throughout the pandemic. Twelve adolescents showed worsening of the symptoms of the underlying disease, increasing to twenty-two after 12 months. Fifteen patients reported COVID-19 infection with mild symptoms. Nineteen patients reported that a family member had contracted the disease. Only 18 participants returned to in-person school, with the remainder preferring to maintain remote learning.
Ferro et al., 2021 [34] Canada To describe changes in psychological stress during the COVID-19 pandemic in adolescents with chronic diseases.	Longitudinal cohort study. Psychological suffering, the impact of COVID-19, the stress of pandemic restrictions, and the impact of these on social relationships and mental health were assessed using various instruments. Study population: 43 teenagers aged 13 to 16 years old with chronic diseases such as asthma, type 1 diabetes, and epilepsy.	Participants reported increased psychological stress compared to the period before the pandemic, mainly related to worry and the effects of social isolation. Teenagers tended to have their stress levels underestimated by their parents. Females had higher levels of stress related to the severity of the underlying disease, psychological distress prior to the pandemic, concerns about the pandemic, effects of social isolation, and less belief that the pandemic would end soon.
Gillon-Keren et al., 2022 [35] Israel To examine whether eating disorders (ED) were exacerbated in adolescents with type 1 diabetes during the COVID-19 pandemic and whether changes in glycemic control and eating habits occurred.	Longitudinal observational study with a quantitative approach. Instruments were applied to assess the risk of eating disorders (ED) and the quality of the diet. The data were compared to the pre-pandemic period. Study population: 34 adolescents aged 15 to 19 years old diagnosed with T1D.	The lockdown negatively affected the eating behaviors of the adolescents, doubling the likelihood of having eating disorders (ED). The pandemic did not affect glycemic control, body mass index, or adherence to the Mediterranean diet (eating plan emphasizing healthy fats, whole grains, fruits, vegetables, beans, nuts, and seeds). Social restrictions imposed by COVID-19 and altered sleep patterns may explain the increase in ED.
Helito et al., 2021 [36] Brazil To evaluate sleep quality in adolescents with chronic immunosuppressive diseases during the COVID-19 pandemic.	Cross-sectional study with a quantitative approach. General information was collected about COVID-19, the impact of quarantine and physical health during the pandemic, sleep quality and disorders, and quality of life and school functionality. Data collection was conducted through instruments. Study population: 387 adolescents aged 10 to 18 years old, including 305 diagnosed with conditions such as juvenile idiopathic arthritis, systemic lupus, dermatitis, kidney conditions, inflammatory bowel disease, celiac disease, autoimmune hepatitis, and liver transplant recipients, and 82 healthy peers.	Frequencies of poor sleep quality were similar between healthy adolescents and those with chronic diseases (CD). Adolescents with chronic diseases had lower scores for sleep latency and daytime dysfunction due to sleepiness and higher happiness scores compared to the group of healthy adolescents. Among adolescents with chronic diseases, the highest rate of poor sleep quality was associated with attending a public school, increased screen time, intrafamily violence, and a greater fear of using immunosuppressive medication, mainly among girls. Lower perceived health-related quality of life was also linked to worse sleep quality in this group.
Hoefnagels et al., 2022 [37] Netherlands To assess the impact of the COVID-19 pandemic on the mental well-being of adolescents with chronic conditions, exploring associations between government restrictions and mental well-being.	Longitudinal cohort study. Assessment of mental well-being using three indicators: satisfaction with life, internalization of symptoms, and psychosomatic health. Government restrictions during COVID-19 were also measured. Study population: 477 adolescents aged 12 to 18 years old, including 311 diagnosed with cystic fibrosis, autoimmune disease, heart or kidney disease, and 166 healthy peers.	Life satisfaction was significantly lower after the pandemic, particularly in children with chronic illnesses, especially girls; healthy children also showed reduced satisfaction. Girls exhibited a higher frequency of internalizing symptoms and psychosomatic symptoms, which were more present in children with chronic illnesses. There was no statistically significant difference in symptoms between the periods before and after the pandemic. Regarding government restrictions, higher rigidity scores were linked to worse life satisfaction, increased internalization of symptoms, and appearance of psychosomatic symptoms.
Karaatmaca et al., 2022 [38] Turkey To evaluate the quality of life (QoL) of adolescents with asthma and the anxiety that may occur due to the COVID-19 pandemic, in addition to asthma control.	Observational and cross-sectional study. Information collected included medications, symptoms, and control of the chronic disease status. Application of questionnaires to identify rates, status, and levels of anxiety; physical and emotional health and functionality; identification of asthma symptoms, impact on daily life, and need for medication in the last four weeks. Study population: 61 adolescents aged 12 to 18 years old diagnosed with asthma and 60 healthy peers.	There was no difference between the anxiety scores of the group with and without asthma. Among adolescents with asthma, the group with a more significant lack of disease control had higher levels of anxiety related to a worse perception of quality of life. Deterioration in asthma control was observed with increasing age in adolescents as they change behavior and become more independent regarding parental care. Uncontrolled asthma reduces patients’ quality of life. Girls had a worse perception of the chronic disease related to exacerbation of asthma symptoms, less contact with the health system, and a need to be more educated on how to use inhalers.
Lindoso et al., 2022 [39] Brazil To evaluate physical and mental health indicators in adolescents with chronic immunocompromise during the COVID-19 pandemic.	Cross-sectional study. A questionnaire was used to evaluate routine health care and general information about COVID-19. The impact of quarantine on mental health was also measured using a scale. Study population: 355 adolescents aged 10 to 18 years old with chronic immunocompromised or immune-mediated conditions (gastrointestinal, hepatic, rheumatological, and renal conditions) and 111 healthy peers.	Girls were most afraid of the complications of their chronic disease in the case of COVID-19 infection. Parents working outside the home harmed adolescents’ psychosocial well-being. Physical activity was a protective factor for mental health. With distance learning, regular school tasks were beneficial to psychosocial functioning. Adolescents were excessively afraid of using immunosuppressant medications, believing they increased vulnerability to COVID-19 infection. The closure of public places and remote learning reduced physical performance and sleep quality. There was a reduction in mental health problems in adolescents with chronic illnesses, such as reduced educational stress, increased time with family, reduced access to legal and illicit drugs, and easier access to healthcare through online consultation. Ten percent of patients presented with mental disorders after the health crisis. Those with pre-existing psychopathologies are at greater risk of worsening their mental health, with reduced exposure to sunlight and prolonged daytime sleepiness contributing to worse sleep quality and insomnia.
Logan et al., 2022 [40] Canada To understand how lockdown affected mental health and physical activity in children with neuro-inflammatory disorders.	Longitudinal quantitative study. Scales were applied to assess sleep habits, depression, anxiety, and physical activities. Study population: 314 adolescents aged 13 to 18 years old with neuro-inflammatory disorders.	Sleep increased significantly in the first 6 months of the COVID-19 lockdown. Across the group, anxiety and depression did not change with the pandemic. Anxiety was lower in teenagers than in pre-teens. Depression remained higher in adolescents than in pre-adolescents at both time points. Physical activity levels did not change with the pandemic compared to pre-pandemic. Anxiety was more significant in inactive individuals, regardless of time.
Mazzolani et al., 2021 [15] Brazil To report the impact of COVID-19 on eating habits and sedentary behavior among adolescents with multiple chronic conditions compared to their healthy peers.	Observational study using a questionnaire to evaluate changes in daily routine and leisure activities during the pandemic, and time spent in sedentary behaviors. Study population: 347 adolescents aged 10 to 18 years old, diagnosed with juvenile idiopathic arthritis, lupus erythematosus, celiac disease, inflammatory bowel disease, nephrotic syndrome, renal disease, and kidney transplant recipients, and 95 healthy peers.	A total of 44.3% of the adolescents reported a lower frequency of consumption of processed foods during the pandemic, while healthy adolescents had an increased consumption of this type of food. A total of 33.8% reported greater consumption of home-cooked foods. A total of 21.7% reported eating more frequently with other people. A total of 14.2% said they had reduced the frequency of eating with others during the pandemic. A total of 32.2% of adolescents with chronic diseases reported eating more in front of the television than before the pandemic, compared to 17.4% who reduced this habit. A total of 35.8% of patients said they participated more in preparing meals than before the pandemic. Regarding sedentary habits, there was an increase in screen time, although this was more frequent among healthy adolescents; 44.6% of adolescents with chronic diseases spent more than 6 h in front of a screen.
Mianowska et al., 2021 [41] Poland To compare diabetes-related distress in young patients with T1D and their parents before and during the COVID-19-related national lockdown when schools operated online.	Cohort study. Instruments were applied to assess distress about chronic diseases (CD). Study population: 55 adolescents aged 12 to 18 years old diagnosed with Type 1 diabetes mellitus (DM1).	Before the pandemic, high distress scores were related to increased BMI in adolescents; adolescent girls had higher distress scores compared to boys; the device used to control blood glucose did not change diabetes-related distress scores. During the pandemic, there was a decrease in the distress score, especially among girls between 12 and 18 years old, related to more time at home; 12 adolescents began using continuous glucose monitoring to control blood glucose levels; for these adolescents, there was no worsening of distress scores.
Miller et al., 2022 [42] United States of America To compare youth and parents’ emotional functioning and management of type 1 diabetes after the pandemic about family stress and youth self-regulation.	Observational study and clinical trial with a quantitative approach. Instruments assessed emotional functioning, chronic disease-related stress, and difficulties in regulating emotions. Study population: 43 adolescents aged 13 to 17 years old diagnosed with T1D.	Youth self-regulation moderated changes in self-reported diabetes-related distress from before to during the pandemic. Calming strategies included using emotional awareness, mindfulness, and relaxation strategies to increase emotional self-regulation. Those who used these strategies reported decreased diabetes-related distress. Those with weaker emotional self-regulation reported increased diabetes-related distress.
Passanisi et al., 2020 [43] Italy Investigating behavioral responses during quarantine due to the COVID-19 outbreak in a cohort of Italian pediatric patients with type 1 diabetes (T1D).	Cross-sectional, quantitative cohort study. A questionnaire was used to assess changes in lifestyle and the impact of confinement. Study population: 204 participants aged 5 to 18 years old * diagnosed with T1D, with a focus on adolescents aged 12 to 18 years old.	Many participants could comply with T1D control, avoiding overeating during quarantine, mainly by excluding extra meals that occurred due to school. They were able to do regular physical activities, even at home, which helped to achieve reasonable glycemic control, better lipid profile, body mass control, and well-being. Glycemic monitoring during lockdown decreased in only 18.6% of patients. Online classes and remote contact with colleagues helped minimize negative emotions caused by social isolation.
Taheri et al., 2022 [44] Iran Explore the experiences of Iranian adolescents with cystic fibrosis during the COVID-19 pandemic about coughing.	Qualitative study. Conducted through semi-structured face-to-face interviews. Analyzed using inductive thematic analysis. Study population: 21 teenagers between 12 and 19 years old diagnosed with cystic fibrosis.	Adolescents reported that coughing is the strongest characteristic of their illness, which interferes with their sleep routine and hygiene. Coughing was considered a social stigma of contagious diseases for this group. Adolescents reported that, for fear of contracting COVID-19, they were following prevention measures. They rarely left the house, and they felt upset when their cough was mistaken for a symptom of COVID-19. Teenagers reported that after the pandemic began, friends, family, and others sometimes distanced themselves from them or covered their mouths when they started coughing. For boys, coughing also comes with the stigma of drug addiction and cigarette consumption.
Telford et al., 2021 [45] New Zealand To assess physical activity habits in adolescents with type 1 diabetes (T1D) compared to their healthy peers during the first wave of COVID-19 in New Zealand.	Cross-sectional study with a quantitative approach. Questionnaires were used to assess socioeconomic losses and physical activity levels. Study population: 33 adolescents aged 11 to 18 years old diagnosed with T1D, and 34 healthy peers.	A total of 45% of adolescents with T1D were classified as overweight or obese based on the body mass index (BMI), with 12% in the obesity category. The diagnosis of T1D was correlated with an increase in BMI during the pandemic. Adolescents with T1D, worse economic conditions, and higher BMI tended to have increased glycated hemoglobin levels. During the pandemic, participants showed a low to moderate level of physical activity. Younger adolescents tended to move more than older ones. Adolescents with higher BMI tended to engage in physical activity less frequently. Common activities included walking, cycling, dancing, running, and gymnastics. Those who practiced sports such as dance, gymnastics, martial arts, and basketball spent more time practicing these activities. Girls tended to be more active at home than boys, carrying out activities such as dancing, gymnastics, and aerobics.
Troncone et al., 2022 [46] Italy To explore the prevalence of eating disorder symptoms in patients with type 1 diabetes (T1D) during and after the lockdown period in Italy to assess the changes in symptoms with the relaxation of distancing measures.	Cross-sectional study with a quantitative approach. Application of questionnaires to assess eating disorders. Study population: 85 children and adolescents aged 8 to 19 years *, diagnosed with T1D, and 176 healthy peers.	Among adolescents with T1D, there were no significant changes in glycated hemoglobin levels and body mass index values. Throughout the pandemic, eating disorder assessment scores showed a reduction in oral control, which indicates eating self-control, with girls presenting higher scores than boys. There was an increase in diet scores in the group with T1D, indicating more significant concerns about weight and calorie consumption, especially in adolescents over 13 years of age. In contrast, the same group of healthy peers had higher scores on the bulimia subscale.
Yanaz et al., 2022 [47] Turkey To assess the effect of the COVID-19 pandemic on the family environment and relationships, self-care, peer relationships, psychological reactions including anxiety, and abilities of patients to cope with cystic fibrosis compared to healthy patients.	Cross-sectional, case-control study with a quantitative approach. Behavioral questionnaires were applied, based on psychiatric assessment exams, and separated by age groups, evaluating family environment and relationships, self-care practices and relationships with peers during lockdown, psychological reactions to the COVID-19 pandemic, following news related to COVID-19, and anxiety related to taking tests for admission to high school or university. Study population: 132 patients aged 7 to 18 years old *, diagnosed with cystic fibrosis (presenting pancreatic insufficiency, colonization by Pseudomonas Aeruginosa, and severe lung disease), of which 61 were adolescents and 135 healthy patients.	There was no statistically significant difference between the severity of cystic fibrosis (CF) and the patients’ anxiety levels. Overall, participants with CF showed lower levels of anxiety related to the risk of infection from family members and the pandemic in general. Adolescents with CF communicated more with their friends through social networks. They were upset about the closure of schools, although they were not reluctant to transition activities to an online platform. They experienced changes related to their appetite and sleep patterns, sought fewer activities to reduce anxiety and fewer new hobbies, reported a greater frequency of the feeling of spending enough time with their parents, and presented, to a lesser extent, psychosomatic feelings of pain, weakness, and fatigue. Adolescents also reported high levels of anxiety related to taking school and university entrance exams, with slightly higher levels in healthy adolescents than in those with CF.
Di Dalmazi et al., 2020 [48] Italy To investigate continuous glucose monitoring in children, adolescents, and adults with type 1 diabetes (T1D) during the COVID-19 pandemic and identify possible related factors.	Quantitative study. Data collected included blood glucose values through continuous monitoring 20 days before and 20 days after lockdown and a scale to assess physical activity levels. Study population: 188 patients with T1D, 24 of whom were teenagers aged 13 to 17 years old.	Regarding physical activity, adolescents showed significantly lower levels of moderate physical activity during the pandemic, although overall levels were similar between different age groups. Regarding glucose monitoring, adolescents showed similar measurement frequencies before and during the lockdown, with more excellent stability in blood glucose values.
Campos et al., 2023 [16] Brazil To evaluate the factors associated with emotional changes, hyperactivity, and inattention caused by social isolation due to COVID-19 in patients with immunosuppressive diseases.	Cross-sectional study with a quantitative approach. Application of online forms, including questions about educational data, care routines, sleep, physical activity, information about the pandemic, and impacts of social isolation. Instruments were also used to assess mental health, sleep quality, and quality of life. Study population: 343 adolescents aged 10 and 18 years old with chronic immunosuppressive diseases such as rheumatoid arthritis, kidney disease, liver and gastrointestinal diseases, and 108 healthy adolescents.	Adolescents with chronic diseases (CD) presented worse scores related to mental health, although similar to the results of their healthy peers. Among adolescents with CD and worse mental health, there was a worsening in sleep quality. A higher rate of adolescents enrolled in public schools and who cared for an older relative was noted, showing less frequency of carrying out school tasks and reduced hours of sleep (less than 8 h). Patients with CD also had worse quality of life scores, mainly in the physical and psychosocial health domains, related to worse school performance. Higher frequencies were observed in this group of adolescents for screen time, exposure to family violence, fear of changes in medical monitoring, fear of contracting COVID-19, and complications related to underlying diseases.
Thomas et al., 2023 [49] United States of America To gather perspectives from youth of color, their caregivers, and healthcare providers on telehealth for type 1 diabetes (T1D) care during COVID-19.	Qualitative study. Demographic forms were created to capture race, ethnicity, age, and sex. Semi-structured interview guides were created for youth, caregivers, and healthcare providers. Study population: 16 caregivers, 8 youth aged 12 to 19 years old with T1D, and 9 healthcare providers.	In the beginning, adolescents and caregivers found teleconsultations dubious and described them as “different” or experienced feelings like anxiety. Adolescents had positive perceptions and felt that the consultations were beneficial. Conversely, one adolescent expressed negative perceptions, describing that teleconsultations take longer than in-person consultations. Some logistical and technical barriers were perceived, such as problems with scheduling appointments and internet instability, which hindered the management of type 1 diabetes.
Noij et al., 2023 [50] Netherlands To evaluate the impact of the pandemic on depression, anxiety, and resilience in Dutch people with cystic fibrosis (PwCF) or primary ciliary dyskinesia (PwPCD) and their caregivers.	Quantitative, longitudinal, questionnaire screening study. Data collection included demographic forms and questions about psychological support. Questionnaires to measure depressive symptoms, anxiety, resilience, and impacts on family life were assessed using the Family Exposure and Impact and Survey for Adolescents and Young Adults (CEFIS). Study population: 10 adolescents aged 12 to 17 years old with cystic fibrosis (CF) (9%) and 9 adolescents with disabilities (8%).	Adolescents with cystic fibrosis (CF) underwent psychological therapy or had done so at some point in the last year. For those with disabilities, 26% had undergone therapy, and 13% required psychological support. Taking this into account, 1 of the adolescents with disabilities presented suicidal ideation. Meanwhile, 6 (5.5%) participants in both the CF and disabilities group showed elevated levels of depression, and 12 (10.9%) showed elevated levels of anxiety. In the analysis of differences between the presence of anxiety and depression among groups, participants with disabilities exhibited higher levels. The resilience scale and CEFIS yielded average results across the sample.
Zucchetti et al., 2023 [51] Italy This study set out to evaluate the psychosocial effects of COVID-19 among adolescents with cancer and whether these effects are significantly different among adolescents who were undergoing therapy or had completed it.	Multicenter national study. Used a 16-item Likert scale to assess the frequency of feelings related to COVID-19. Study population: 214 adolescents aged 15 to 19 years diagnosed with leukemia and lymphoma (47%), bone sarcoma (20%), solid tumors (18%), or brain tumors (15%).	The participants expressed fear for others, particularly for family and friends, equally distributed within the group, without significantly impacting routine and sleep during the COVID-19 pandemic. For 46.7%, personal protective items and social distancing did not remind them of treatment periods. A total of 59.2% had no trouble adhering to pandemic restrictions. Those who had completed treatment often disregarded protective measures. With social distancing, participants missed social contact (25.4% increased their use of social media, and 33.7% increased their internet usage for watching series, movies, and playing online games).
Rovira-Remisa et al., 2023 [52] Spain This study aimed to assess the quality of life and to characterize the psychopathological status of patients with rare diseases in Spain, with a particular focus on inherited metabolic disorders (IMD).	A prospective case-control study. Quality of life (QoL) and mental health were assessed using validated scales according to age. Inclusion criteria: All patients above four years of age suffering from a definite rare disease, diagnosed either with a genetic test, a confirmatory biochemical profile, or clinical assessment. Study population: 40 adolescents aged 14 to 17 years old diagnosed with an IMD, such as genetic syndromes, neuromuscular or inherited metabolic disorders, and 48 healthy controls.	Adolescent patients did not show a statistically significant difference in mental status when compared to their control group. Adolescent patients showed worse mental health preservation than children, mainly in the control groups, which showed higher levels of mental distress. Data did not show significant statistical differences when comparing quality of life between cases and controls in children or adolescents.
Tremolada et al., 2023 [53] Italy This study aimed to provide a more in-depth understanding of how the COVID-19 pandemic affected adolescents with type 1 diabetes (T1D) routines, experiences, T1D management, and psychological well-being.	A reflexive thematic analysis, qualitative study. Data collection method: Focus groups. Study population: 24 adolescents (M/F = 17/7) aged 15 to 18 years old diagnosed with type 1 diabetes (T1D) with no restriction on T1D duration.	A total of 33% reported improved glycemic control due to increased self-care, including learning, positivity, and exercise. Better sleep and home-cooked meals were cited. However, restrictions on physical activity, diet changes, and ongoing adjustments to T1D therapy led to worse outcomes. Adolescents did not consider themselves at higher risk for a severe clinical course of COVID-19 when adhering to restrictions, even though they experienced intense emotions. Pandemic-related changes like remote learning and parental presence caused a sense of loss but also heightened appreciation for relationships.
Zeiler et al., 2023 [54] Austria This study aimed to investigate the psychosocial impact of the COVID-19 pandemic on adolescents with type 1 diabetes (T1D).	Qualitative study. Data collection method: 18 semi-structured interviews. Study population: 10 adolescents with T1D aged 14–18 years old diagnosed with T1D for more than 6 months (interviews included both the adolescents and their parents).	Adolescents coped well with the COVID-19 pandemic, especially with reliable information. There were technical issues with their glucose monitoring devices, including allergies to sensor adhesives. Managed to maintain control without additional consultations. Adapted well to online appointments, although they could not assess insulin injection sites. Worries included acquiring and stockpiling supplies and experiencing delivery delays. Being at home allowed for family moments and parental assistance in glucose control. Some adolescents felt more independent, which led to monitoring their diet, reducing school-related stress, and better managing glucose fluctuations. Regarding physical activity, some remained active with alternative exercises; others increased food intake and became sedentary.
Collins et al., 2023 [55] United States of America This study aimed to explore the perceptions of adolescents with poorly controlled asthma regarding their asthma severity and self-management during the pandemic, exploring differences in these beliefs by urbanicity, sex, and race/ethnicity.	Drawn from the baseline data of two clinical trials. Assessment of school-based interventions to improve asthma control among adolescents with uncontrolled asthma. Study population: 183 adolescents with asthma aged 13.0 to 17.9 years old at enrollment (Study 1: Participants aged 13.0 to 17.9 years old; Study 2: Participants aged 9 to 12 years old).	Most (68.4%) reported that their asthma severity remained unchanged; 26.0% reported it worsened. Nearly 30% reported they altered how they managed their asthma, with most (80%) reporting additional efforts. Compared with asthma remaining the same, females had a higher relative risk than males of reporting that their asthma worsened. Urban youth had greater odds of reporting they changed their asthma self-management than rural peers.
Framme et al., 2023 [56] Germany This study aimed to assess the Health-Related Quality of Life (HRQoL) of adolescents with type 1 diabetes since the second wave of the COVID-19 pandemic.	Anonymous cross-sectional survey, conducted at three German diabetes centers. Teen HRQoL was assessed by using self-report and parent-proxy reports. Study population: 45 adolescents with type 1 diabetes aged 12 to 18 years old and 413 parents.	Most adolescents reported an average (75.5%) HRQoL. Approximately 11.3% of teens reported high and 13.2% low HRQoL. Teen’s female gender, older age, higher diabetes burden, and parental depression symptoms contributed to lower self-reported HRQoL among teens. Compared to healthy peers during the first wave of the pandemic, adolescents in the current study reported higher HRQoL.

**Table 2:** COVID-19 impacts and related affecting aspects on adolescents with chronic diseases.

Category	Subcategory	Impacts	Related Affecting Aspects
Reinventing themselves when facing social isolation, school closure, and new family interactions	Changes in social and family interactions	Increased interaction between adolescents with CD and their families [27,29,30]	- Greater adolescent satisfaction [47] - Increased exposure of adolescents to physical and psychological violence by their families [16,33,36]
		Some parents refrained from face-to-face work for fear of contracting the virus and passing it on to their children [31]	- Worsening economic conditions [27,33] - Increased family tension [27,29]
		Some parents kept working/needed to return to work [39]	- Worsening of adolescents’ psychosocial well-being [39]
		Isolation from extended family and friends [39]	- Increased stress between parents and children [33,44]
		Association of adolescents with a means of transmission of COVID-19 [44]	- Increased stigma of some underlying diseases, such as cystic fibrosis, mainly due to the association between its symptoms and the symptoms of COVID-19 [44]
	Educational adaptations and the use of technology	Remote learning [47]	- Increased negative emotions associated with the breakdown of normality [43] - Increased psychosocial functioning of adolescents [39,47] - Reduced quality of learning among adolescents with CD [16]
		Increased use of technology [27]	- Increased screen usage (social media/study time) [28,36] - Sense of security [31]
		Resistance to returning to classroom learning [26,31,33]	- Fear of jeopardizing their health [26,31,33]
	Psychological and emotional changes	Adolescents with CD were less psychologically affected than their healthy peers [36,39,40]	- Reduced anxiety about the possibility of contamination [38] - Good level of adolescents’ knowledge of COVID-19 [21]
		Adolescents’ lack of prospects for the future, especially about financial and school issues [33,47]	- Increased anxiety in the first 12 months of the pandemic [33,47] - Girls were more affected [38,39]
		The worse overall perception of quality of life [31,39]	- Increased psychosomatic symptoms such as anxiety and stress [27,39,47] - Lack of information on the specific effects of COVID-19 in adolescents with CD [31]
		Belief of greater vulnerability to infection by the COVID-19 virus [27,30,34,36]	- Greater frequency of feelings of anguish [27,30,34,36]
Chasing for the best decision: following up the chronic disease while fighting the COVID-19 pandemic	Influence of socioeconomic status on access to health services	Adolescents relying solely on public healthcare services faced exacerbated challenges in managing chronic diseases [24,29,31,45]	- Access to vital healthcare resources, including continuous monitoring devices, became limited [29] - Worsened disease control and symptoms [38,41]
		Adolescents with private medical–hospital insurance experienced improved disease management [24,32,41]	- Telemedicine [28,29,32] - Maintained access to control devices [24,28,29,32,41]
	New usual care routine related to CD	Less ability to effectively manage chronic diseases [28,29,43]	- Socioeconomic vulnerabilities, including housing insecurity and income instability [28,29,43] - Adolescents who self-managed their care without parental supervision [28,32,38,45]
		Decreased healthcare continuity [22,40,45]	- Fears of contracting COVID-19 deterred adolescents and their families from seeking in-person medical follow-up [26] - Misinformation about COVID-19 and its interaction with specific medications used in chronic disease’s usual treatment [26,27]
		Maintenance or increase in monitoring of signs and symptoms [24,25,29,30,43,46,48]	- Adolescents who had parental supervision at home adhered more easily to treatment [25,29,30] - Adolescents’ belief that DC could be a risk factor for a worse COVID-19 prognosis [30]
	Changes in lifestyle habits and their consequences	Adoption of self-management practices [42]	- Adherence to treatment and better overall health [42]
		Maintenance of physical activity levels and practicing healthy nutrition [30,35,40,46]	- Increased well-being [39] - Girls were more active [45]
		Changes in the sleep patterns, increased sedentary behavior, and changes in eating patterns [27,28,35,38,39,40,42,47]	- Prolonged isolation [31,39] - Increased psychosomatic symptoms such as anxiety and stress [31,39] - Worse overall perception of quality of life [31,39]
		Adolescents with CD demonstrated poorer physical health compared to healthy peers [16]	- Notable reduction in physical activity [28,32]
		Worsening of some symptoms in chronic skin conditions [26,31]	- COVID-19 preventive measures like the use of hand sanitizers and masks [26,31]
		Good adaptation to COVID-19 prevention measures [27]	- Frequent handwashing [27,34,44]

## Data Availability

The original contributions presented in the study are included in the article/Supplementary materials, further inquiries can be directed to the corresponding author/s.

## Supplementary Materials

The following supporting information can be downloaded at https://www.mdpi.com/article/10.3390/children11091047/s1, Supplementary File S1.
