# Supplementary material for: Navigating the Challenges and Resilience in the Aftermath of the COVID-19 Pandemic in Adolescents with Chronic Diseases: A Scoping Review
Source: Children (Basel). 2024 Aug 27;11(9):1047. doi: 10.3390/children11091047 (PMC11430041; doi:10.3390/children11091047)
Supplement: Supplementary file 1 [file children-11-01047-s001.zip › children-3171109-supplementary.pdf]

Supplementary File S1: Search strategies in the databases.

LILACS: (ADOLESCENTE) AND (DOENÇAS CRONICAS) AND (COVID-19)

PUBMED: ((("Adolescent"[Mesh] OR (Adolescents) OR (Adolescence) OR (Teens) OR (Teen) OR (Teenagers) OR (Teenager)) AND ("Chronic Disease"[Mesh] OR (Chronic Diseases) OR (Disease, Chronic) OR (Diseases, Chronic) OR (Chronic Illness) OR (Chronic Illnesses) OR (Illness, Chronic) OR (Illnesses, Chronic) OR (Chronically Ill) OR "Diabetes Mellitus, Type 1 "[Mesh] OR (Diabetes Mellitus, Type I) OR (Type 1 Diabetes Mellitus) OR (Diabetes Mellitus, Insulin-Dependent, 1) OR (Insulin-Dependent Diabetes Mellitus 1) OR (Insulin Dependent Diabetes Mellitus 1) OR (Type 1 Diabetes) OR (Diabetes, Type 1) OR (Diabetes Mellitus, Insulin-Dependent) OR (Diabetes Mellitus, Insulin Dependent) OR (Insulin-Dependent Diabetes Mellitus) OR (Neoplasia) OR (Neoplasias) OR (Neoplasm) OR (Tumors) OR (Tumor) OR (Cancer) OR (Cancers) OR (Malignant Neoplasms) OR (Malignant Neoplasm) OR (Neoplasm, Malignant) OR (Neoplasms, Malignant) OR (Malignancy) OR (Malignancies) OR "Asthma"[Mesh] OR (Asthmas) OR (Bronchial Asthma) OR (Asthma, Bronchial) OR "Renal Insufficiency, Chronic "[Mesh] OR "Kidney Failure, Chronic"[Mesh] OR (Chronic Renal Insufficiencies) OR (Renal Insufficiencies, Chronic) OR (Chronic Renal Insufficiency) OR (Kidney Insufficiency, Chronic) OR (Chronic Kidney Insufficiency) OR (Chronic Kidney Insufficiencies) OR (Kidney Insufficiencies, Chronic) OR (Chronic Kidney Diseases) OR (Chronic Kidney Disease) OR (Disease, Chronic Kidney) OR (Diseases, Chronic Kidney) OR (Kidney Disease, Chronic) OR (Kidney Diseases, Chronic) OR (Chronic Renal Diseases) OR (Chronic Renal Disease) OR (Disease, Chronic Renal) OR (Diseases, Chronic Renal) OR (Renal Disease, Chronic) OR (Renal Diseases, Chronic) OR Cystic Fibrosis"[Mesh]" OR (Fibrosis, Cystic) OR (Mucoviscidosis) OR (Pulmonary Cystic Fibrosis) OR (Cystic Fibrosis, Pulmonary) OR (Pancreatic Cystic Fibrosis) OR (Cystic Fibrosis, Pancreatic) OR (Fibrocystic Disease of Pancreas) OR (Pancreas Fibrocystic Disease) OR (Pancreas Fibrocystic Diseases) OR (Cystic Fibrosis of Pancreas) OR Gastrointestinal Diseases"[Mesh]" OR (Disease, Gastrointestinal) OR (Diseases, Gastrointestinal) OR (Gastrointestinal Disease) OR (Functional Gastrointestinal Disorders) OR (Disorder, Functional Gastrointestinal) OR (Disorders, Functional Gastrointestinal) OR (Functional Gastrointestinal Disorder) OR (Gastrointestinal Disorder, Functional) OR (Gastrointestinal Disorders, Functional) OR "Heart Diseases"[Mesh] OR (Disease, Heart) OR (Diseases, Heart) OR (Heart Disease) OR (Cardiac Diseases) OR (Cardiac Disease) OR (Disease, Cardiac) OR (Diseases, Cardiac))) AND ("COVID-19"[Mesh] OR (COVID 19) OR (COVID-19 Pandemic) OR (Pandemic, COVID-19) OR (COVID-19 Pandemics) OR "Quarantine"[Mesh] OR (Quarantines) OR (Stay at Home Orders) OR (Lockdown, Health) OR (Health Lockdown) OR (Health Lockdowns) OR (Lockdowns, Health) OR (Cordon Sanitaire))) AND ("Health Behavior"[Mesh] OR "Life Change Events"[Mesh] OR (Experience) OR (Experiences) OR (Sense) OR (Senses) OR (Meaning) OR (Meanings) OR (Life Change Event) OR (Life Experience) OR (Experience, Life) OR (live experience) OR (perspective) OR "Life Style"[Mesh] OR (Life Styles) OR (Lifestyle) OR (Lifestyles) OR (Lifestyle Factors) OR (Factor, Lifestyle) OR (Lifestyle Factor) OR "Quality of Life"[Mesh] OR (Life Quality) OR (Health-Related Quality Of Life) OR (Health Related Quality Of Life) OR "Quality of Life"[Mesh] OR (Life Quality) OR (Health-Related Quality Of Life) OR (Health Related Quality Of Life) OR "Adaptation, Psychological"[Mesh] OR (Adaptation, Psychologic)

OR (Psychologic Adaptation) OR (Psychological Adaptation) OR (Adjustment) OR (Coping Behavior) OR (Behavior, Coping) OR (Behaviors, Coping) OR (Coping Behaviors) OR (Coping Skills) OR (Coping Skill) OR (Skill, Coping) OR (Skills, Coping) OR (Coping Strategies) OR (Coping Strategy)OR (Strategies, Coping) OR (Strategy, Coping) OR (Behavior, Adaptive) OR (Adaptive Behavior) OR (Adaptive Behaviors) OR (Behaviors, Adaptive) OR (Instrument) OR (Tools) OR (changes) OR (challenges) OR (Impact) OR (Emotions) OR (Perspectives) OR (Evaluation) OR (Health vulnerability)OR (Feelings)) – FILTERS (YEAR AND LANGUAGE)

SCOPUS: (TITLE-ABS-KEY (("Adolescence") OR "adolescent" OR "Teens" OR "Teenagers" OR "Teenager" OR "Teen") AND TITLE-ABS-KEY (("Chronic Disease") OR "Chronic Diseases" OR "Disease, Chronic" OR "Diseases, Chronic" OR "Chronic Illness" OR "Chronic Illnesses" OR "Illnesses, Chronic" OR "Chronically Ill" OR ("Diabetes Mellitus, Type 1") OR "Diabetes Mellitus, Type I" OR "Type 1 Diabetes Mellitus" OR "Diabetes Mellitus, Insulin-Dependent, 1" OR "Insulin-Dependent Diabetes Mellitus 1" OR "Insulin Dependent Diabetes Mellitus 1" OR "Type 1 Diabetes" OR "Diabetes, Type 1" OR "Diabetes Mellitus, Insulin-Dependent" OR "Diabetes Mellitus, Insulin Dependent" OR "Insulin-Dependent Diabetes Mellitus" OR "Neoplasia" OR "Neoplasias" OR "Neoplasm" OR "Tumors" OR "Tumor" OR "Cancer" OR "Cancers" OR "Malignant Neoplasms" OR "Malignant Neoplasm" OR "Neoplasm, Malignant" OR "Neoplasms, Malignant" OR "Malignancy" OR "Malignancies" OR ("Asthma") OR "Asthmas" OR "Bronchial Asthma" OR "Asthma, Bronchial" OR ("Renal Insufficiency, Chronic") OR ("Kidney Failure, Chronic") OR "Chronic Renal Insufficiencies" OR "Renal Insufficiencies, Chronic" OR "Chronic Renal Insufficiency" OR "Kidney Insufficiency, Chronic" OR "Chronic Kidney Insufficiency" OR "Chronic Kidney Insufficiencies" OR "Kidney Insufficiencies, Chronic" OR "Chronic Kidney Diseases" OR "Chronic Kidney Disease" OR "Disease, Chronic Kidney" OR "Diseases, Chronic Kidney" OR "Kidney Disease, Chronic" OR "Kidney Diseases, Chronic" OR "Chronic Renal Diseases" OR "Chronic Renal Disease" OR "Disease, Chronic Renal" OR "Diseases, Chronic Renal" OR "Renal Disease, Chronic" OR "Renal Diseases, Chronic" OR ("Cystic Fibrosis") OR "Fibrosis, Cystic" OR "Mucoviscidosis" OR "Pulmonary Cystic Fibrosis" OR "Cystic Fibrosis, Pulmonary" OR "Pancreatic Cystic Fibrosis" OR "Cystic Fibrosis, Pancreatic" OR "Fibrocystic Disease of Pancreas" OR "Pancreas Fibrocystic Disease" OR "Pancreas Fibrocystic Diseases" OR "Cystic Fibrosis of Pancreas" OR ("Gastrointestinal Diseases") OR "Disease, Gastrointestinal" OR "Diseases, Gastrointestinal" OR "Gastrointestinal Disease" OR "Functional Gastrointestinal Disorders" OR "Disorder, Functional Gastrointestinal" OR "Disorders, Functional Gastrointestinal" OR "Functional Gastrointestinal Disorder" OR "Gastrointestinal Disorder, Functional" OR "Gastrointestinal Disorders, Functional" OR ("Heart Diseases") OR "Disease, Heart" OR "Diseases, Heart" OR "Heart Disease" OR "Cardiac Diseases" OR "Cardiac Disease" OR "Disease, Cardiac" OR "Diseases, Cardiac") AND TITLE-ABS-KEY (("COVID-19") OR ("COVID-19 Pandemic") OR ("Quarantine") OR ("Stay-at-Home Orders") OR "Stay at Home Orders") AND TITLE-ABS-KEY (("Life Experiences") OR "Experience" OR "sense" OR "Senses" OR "Meaning" OR "Meanings" OR "Life Change Event" OR "Life Experience" OR "Experience, Life" OR "live experience" OR "perspective" OR ("Life Style Changes") OR ("Quality of Life") OR ("Adaptation, Psychological") OR "Adjustment" OR "Coping Behavior" OR "Behavior, Adaptive" OR "Instrument" OR "Tools" OR "changes" OR "challenges" OR "Impact" OR ("Emotions") OR "Perspectives" OR ("Evaluation") OR "Health vulnerability" OR ("Life Course Perspective"))) AND PUBYEAR = 2023 AND (LIMIT-TO (LANGUAGE ,

"English") OR LIMIT-TO (LANGUAGE , "Portuguese") OR LIMIT-TO (LANGUAGE , "Spanish"))

CINAHL - ((MH "Adolescence") OR "adolescent" OR "Teens" OR "Teenagers" OR "Teenager" OR "Teen") AND ((MH "Chronic Disease") OR "Chronic Diseases" OR "Disease, Chronic" OR "Diseases, Chronic" OR "Chronic Illness" OR "Chronic Illnesses" OR "Illnesses, Chronic" OR "Chronically Ill" OR (MH "Diabetes Mellitus, Type 1") OR "Diabetes Mellitus, Type I" OR "Type 1 Diabetes Mellitus" OR "Diabetes Mellitus, Insulin-Dependent, 1" OR "Insulin-Dependent Diabetes Mellitus 1" OR "Insulin Dependent Diabetes Mellitus 1" OR "Type 1 Diabetes" OR "Diabetes, Type 1" OR "Diabetes Mellitus, Insulin-Dependent" OR "Diabetes Mellitus, Insulin Dependent" OR "Insulin-Dependent Diabetes Mellitus" OR "Neoplasia" OR "Neoplasias" OR "Neoplasm" OR "Tumors" OR "Tumor" OR "Cancer" OR "Cancers" OR "Malignant Neoplasms" OR "Malignant Neoplasm" OR "Neoplasm, Malignant" OR "Neoplasms, Malignant" OR "Malignancy" OR "Malignancies" OR (MH "Asthma") OR "Asthmas" OR "Bronchial Asthma" OR "Asthma, Bronchial" OR (MH "Renal Insufficiency, Chronic") OR (MH "Kidney Failure, Chronic") OR "Chronic Renal Insufficiencies" OR "Renal Insufficiencies, Chronic" OR "Chronic Renal Insufficiency" OR "Kidney Insufficiency, Chronic" OR "Chronic Kidney Insufficiency" OR "Chronic Kidney Insufficiencies" OR "Kidney Insufficiencies, Chronic" OR "Chronic Kidney Diseases" OR "Chronic Kidney Disease" OR "Disease, Chronic Kidney" OR "Diseases, Chronic Kidney" OR "Kidney Disease, Chronic" OR "Kidney Diseases, Chronic" OR "Chronic Renal Diseases" OR "Chronic Renal Disease" OR "Disease, Chronic Renal" OR "Diseases, Chronic Renal" OR "Renal Disease, Chronic" OR "Renal Diseases, Chronic" OR (MH "Cystic Fibrosis") OR "Fibrosis, Cystic" OR "Mucoviscidosis" OR "Pulmonary Cystic Fibrosis" OR "Cystic Fibrosis, Pulmonary" OR "Pancreatic Cystic Fibrosis" OR "Cystic Fibrosis, Pancreatic" OR "Fibrocystic Disease of Pancreas" OR "Pancreas Fibrocystic Disease" OR "Pancreas Fibrocystic Diseases" OR "Cystic Fibrosis of Pancreas" OR (MH "Gastrointestinal Diseases") OR "Disease, Gastrointestinal" OR "Diseases, Gastrointestinal" OR "Gastrointestinal Disease" OR "Functional Gastrointestinal Disorders" OR "Disorder, Functional Gastrointestinal" OR "Disorders, Functional Gastrointestinal" OR "Functional Gastrointestinal Disorder" OR "Gastrointestinal Disorder, Functional" OR "Gastrointestinal Disorders, Functional" OR (MH "Heart Diseases") OR "Disease, Heart" OR "Diseases, Heart" OR "Heart Disease" OR "Cardiac Diseases" OR "Cardiac Disease" OR "Disease, Cardiac" OR "Diseases, Cardiac") AND (MH "COVID-19") OR (MH "COVID-19 Pandemic") OR (MH "Quarantine") OR (MH "Stay-at-Home Orders") OR "Stay at Home Orders" AND (MH "Health Behavior") OR (MH "Life Change Events") OR (MH "Life Experiences") OR "Experience" OR "sense" OR "Senses" OR "Meaning" OR "Meanings" OR "Life Change Event" OR "Life Experience" OR "Experience, Life" OR "live experience" OR "perspective" OR (MH "Life Style Changes") OR (MH "Quality of Life") OR (MH "Adaptation, Psychological") OR "Adjustment" OR "Coping Behavior" OR "Behavior, Adaptive" OR "Instrument" OR "Tools" OR "changes" OR "challenges" OR "Impact" OR (MH "Emotions") OR "Perspectives" OR (MH "Evaluation") OR "Health vulnerability" OR (MH "Life Course Perspective") FILTERS (YEAR AND LANGUAGE))
